# Driving simulator scenarios and measures to faithfully evaluate risky driving behavior: A comparative study of different driver age groups

**DOI:** 10.1371/journal.pone.0185909

**Published:** 2017-10-10

**Authors:** Jesse Michaels, Romain Chaumillon, David Nguyen-Tri, Donald Watanabe, Pierro Hirsch, Francois Bellavance, Guillaume Giraudet, Delphine Bernardin, Jocelyn Faubert

**Affiliations:** 1 Visual Psychophysics and Perception Laboratory, School of Optometry, Université de Montréal, Montréal, Quebec, Canada; 2 Virage Simulation, Montréal, Canada; 3 Interuniversity Research Centre on Enterprise Networks, Logistics and Transportation (CIRRELT) and Department of Management Sciences, HEC Montréal, Montréal, Canada; 4 Essilor International, R&D, Paris, France; 5 Essilor Canada Ltd., Montréal, Quebec, Canada; Beihang University, CHINA

## Abstract

To investigate the links between mental workload, age and risky driving, a cross-sectional study was conducted on a driving simulator using several established and some novel measures of driving ability and scenarios of varying complexity. A sample of 115 drivers was divided into three age and experience groups: young inexperienced (18–21 years old), adult experienced (25–55 years old) and older adult (70–86 years old). Participants were tested on three different scenarios varying in mental workload from low to high. Additionally, to gain a better understanding of individuals’ ability to capture and integrate relevant information in a highly complex visual environment, the participants’ perceptual-cognitive capacity was evaluated using 3-dimensional multiple object tracking (3D-MOT). Results indicate moderate scenario complexity as the best suited to highlight well-documented differences in driving ability between age groups and to elicit naturalistic driving behavior. Furthermore, several of the novel driving measures were shown to provide useful, non-redundant information about driving behavior, complementing more established measures. Finally, 3D-MOT was demonstrated to be an effective predictor of elevated crash risk as well as decreased naturally-adopted mean driving speed, particularly among older adults. In sum, the present experiment demonstrates that in cases of either extreme high or low task demands, drivers can become overloaded or under aroused and thus task measures may lose sensitivity. Moreover, insights from the present study should inform methodological considerations for future driving simulator research. Importantly, future research should continue to investigate the predictive utility of perceptual-cognitive tests in the domain of driving risk assessment.

## 1. Introduction

Early versions of driving simulators date back to 1934 [1] but their practicality has been limited. Recent advances in technology allow researchers and therapists access to a new generation of affordable, realistic and sophisticated simulators. As a result, investigations into driving behavior have significantly increased. Compared to on-road driving studies, the virtual environment of a driving simulator provides several advantages. Chief among these is that participants’ reactions to potentially life-threatening driving situations can be evaluated in perfect safety. Driving simulators allow researchers to reliably control, standardize and replicate specific driving events and conditions, such as route difficulty, traffic, weather, in ways that are simply not possible with on-road study designs that use open (i.e. public roads) or closed roads (specially designed closed circuits) [2,3]. Moreover, driving simulators allow researchers to collect and process a wealth of objective, performance-based data in a relatively short time. Despite these many advantages, designing driving-simulator based studies is not without its challenges. In the context of flight simulators, Blickensderfer et al. [4] showed that correctly-designed scenarios as well as appropriate performance measurements were critical for proper implementation of simulations for training and research. More recently, Matas et al. [5] suggested that driving simulator validity was highly dependent on the specific population under study and the scenarios selected (see also Mullen et al. [6]).

Regarding specific driving populations, two specific age groups have principally been investigated. Studies have been done to develop reliable simulator-based driving assessments for older drivers [7,8,9]. Driving simulators have also been used to develop the best means of helping young drivers attain automaticity in their basic vehicle control skills [10]. Despite driving less on average, elderly drivers are known to be involved in more lethal crashes and have more traffic convictions as compared to any other adult age group [11,12,13]. Furthermore, young drivers are known to be involved in the greatest amount of accidents as compared with any other age group [14]. This makes sense considering their relative inexperience as well as their propensity to take greater risks while driving [15]. Exact reasons notwithstanding, the observed crashes among younger and older drivers cannot be attributed to the same root causes but both may be linked, in part, to difficulties in managing driving situations under high cognitive workload [16]. What is not well understood, however, is how age differences manifest due to variations in mental workload.

When attempting to conceptualize human driving behavior, Keskinen [17] (1996) proposed a four-level, hierarchical model to explain the interplay between different elements of driving skills across levels (see also Hatakka et al. [10]). The importance of the two higher levels that concern social and personality traits (*i*.*e*. “goals for life and skills for living” and “goals and context of driving”) is not obvious in the context of virtual reality. However, driving simulators seem to be ideal tools for assessing the relationship between the two lower levels (*i*.*e*. “mastering traffic situations” and “vehicle maneuvering”). Until recently, most driving simulator studies focused on single measures of “vehicle maneuvering” such as mean driving speed, direction and lane position [18]. Following the increased awareness that single measures, *e*.*g*. mean driving speed, are insufficient criteria to evaluate risky driving behavior [19], there has been renewed interest in the “mastering traffic situations” level. In line with this idea, some studies investigated the link between functional (*e*.*g*. cognitive abilities such as attention) and driving measures (*e*.*g*. mean speed) to assess the potential influence of “mastering traffic situations” on “vehicle maneuvering” level [20–23]. The striking correlations between cognitive ability and driving measures found in these studies reinforce the idea that vehicle driving in traffic is a complex task involving multiple cognitive and perceptual processes such as attention, working memory and executive functioning [24]. These relationships are further demonstrated by a growing body of literature on the aging process that demonstrate that decrements in perceptual and cognitive abilities may be associated with decreased driving performance in healthy, aged drivers [25, 3, 26].

Studies of the aging process and driving also investigated to what extent specific cognitive functions predict driving ability. In such paradigms, cognitive functions were assessed through multiple neuropsychological tests and driving measures were recorded throughout a single drive, in predefined and similar conditions. Nevertheless, it is well known that human performance is also dependent on the task demands or mental workload, within each ecological context [27]. Individuals have a limited cognitive capacity [28]. When resource demands exceed resource availability, performance can be impaired [27]. Mental workload increases with driving complexity [29,30]. Therefore, when considering the context of a driving study, scenarios need to be designed with an appropriate level of difficulty and mental workload to identify subtle differences in driving behavior. For example, a driving scenario that is not sufficiently challenging might not detect differences in driving performance measures. Conversely, driving scenarios that are too difficult or that present unrealistic driving events might create excessively high mental loads that do not reflect natural driving behavior. To date, few studies have investigated the scenario characteristics that affect mental workload during driving. Steyvers & De Waard [31] and Cnossen et al. [32] investigated the influence of roadway characteristics and driving speed, respectively, on subjective as well as physiological measures of mental workload. These studies show that increasing task complexity affects mental workload and can negatively influence driving ability. We are unaware of any attempts to analyze and classify the driving conditions associated with mental workloads appropriate for the dual purposes of eliciting realistic behavior in challenging circumstances and making valid inter-individual comparisons of driving behavior. For instance, despite evidence that older drivers compensate for age-related increases in response time by adopting slower speeds [2], the basic question about whether an individual’s driving speed should be tightly controlled in cross-sectional research remains unanswered.

In the present experiment, we assessed the influence of mental workload on driving measures between different age groups by manipulating the situation complexity in distinct simulator scenarios, each one representing a different driving environment with a different mental workload. Additionally, we used a psychophysical task known as 3-Dimensional Multiple Object Tracking (3D-MOT) to link an individual’s ability to capture and integrate relevant information in a highly complex visual environment [33, 34] to measures of driving performance [35] under different mental workloads.

## 2. Material and methods

### 2.1 Participants

A total of 115 licensed drivers (27 women) between the ages of 18 and 86 (mean = 50.28 ± 25.52 (SD) years old) were recruited from the Université de Montréal’s School of Optometry during routine visits or else were referred by the Québec driving license and public auto insurance authority, the SAAQ (Société de l’Assurance Automobile du Québec). All participants were healthy and reported normal or corrected-to-normal vision (*i*.*e*. visual acuity score of 6/7.5 or better with both eyes in Snellen chart and stereoscopic acuity of 50 seconds of arc or better in Randot test). Participants were free of visual, neurological, musculoskeletal, cardiovascular and vestibular impairments. The study adhered to the tenets of the Declaration of Helsinki (last modified, 2004), all tests and procedures were approved by the ethics committee of the Université de Montréal [Comité d’éthique de la recherché en santé (CERES); certificate N˚ 11-082-CERSS-D] and all volunteers signed forms indicating informed consent. To capture a wide range of driving behaviors, volunteers were separated into three different groups based on their age and level of driving experience. The first group was composed of twenty-nine young adults (5 women), inexperienced drivers (< 1 year of experience driving) ranging in age from 18 to 21 years of age (mean = 20.15 ± 1.19). The second was a group of thirty-five experienced (7 women; ≥ 5 years of experience driving) adults ranging from 25 to 55 years of age (mean = 36 ± 8.68). Finally, the third group consisted of fifty-one experienced (15 women; ≥ 40 years of experience driving) older drivers ranging from 70 to 86 years of age (mean = 77.20 ± 5.01).

### 2.2a Apparatus

A VS500M car driving simulator (Virage Simulation Inc.®) was employed for all driving sessions. This high fidelity, motion-based driving simulator uses real car parts for the cockpit that includes a real car seat, steering wheel, controls, indicators, dashboard and pedals. The steering wheel provides realistic force feedback and the accelerator and brake pedals function as in a typical car. The computerized driving simulation task was displayed under ambient lighting on three 50-inch plasma screens with 1280 x 720-pixel resolution allowing a full 180° field of view. Two additional smaller screens are placed beside and behind the participants to replicate the blind spot areas of the car. Rearview and side mirrors were inset in the central screen to approximate their spatial positions in a real car. The simulation was made even more immersive with motion and sound cues. Realism is enhanced by haptic feedback from a motion system consisting of a compact three-axis platform with electrical actuators that provide acceleration, engine vibration and road texture cues as a function of driving speed. A stereo sound system provides naturalistic engine and external road sounds and the Doppler Effect to recreate the sounds of passing traffic, also as a function of driving speed.

### 2.2b Scenario design

While reviewing the relationship between mental workload and driving, Paxion et al. [36] summarized the taxonomy of situation complexity (see also [18, 37]) as depending on the “road design (*i*.*e*. motorways vs. rural roads vs. city roads), road layout (straight vs. with curves, level vs. inclined, junction vs. no junction) and traffic flow (high density vs. low density)”. Following this, the urban scenario was designed to invoke the sensation of driving in the downtown core of a populated city center and thus involved many more intersections, turns and traffic than the other scenarios. The highway scenario, by comparison, involved fewer of these elements. It was designed to be low in mental loading due to the scarcity of turns, intersections and distracting visual information. Finally, the rural scenario was designed as a middle ground between these two scenarios. The road design, visual information and traffic flows used in these three scenarios led to the following classification from high to low mental workload: urban, rural, and highway.

### 2.3 Protocol

Participants were tested in two experimental sessions separated from each other by a week. Each session lasted approximately one hour. The first session consisted of a visual exam including ETDRS (Early Treatment Diabetic Retinopathy Study) visual acuity testing, Humphrey visual field testing as well as Randot stereoacuity tests meant to screen any drivers with obvious uncorrected visual deficits. The Mini-Mental State Exam (MMSE; Folstein et al. [38]) was also included to screen individuals with strong cognitive impairment. Finally, participants were invited to try the driving simulator in an unrecorded session lasting twelve minutes (two scenarios of six minutes each). This initial introduction to the driving simulator was intended to allow participants to adapt to the simulated environment reducing potentially confounding factors such as Simulator Adaptation Sickness or unfamiliarity with handling the simulator vehicle during the actual testing session [39].

**Fig 1:**
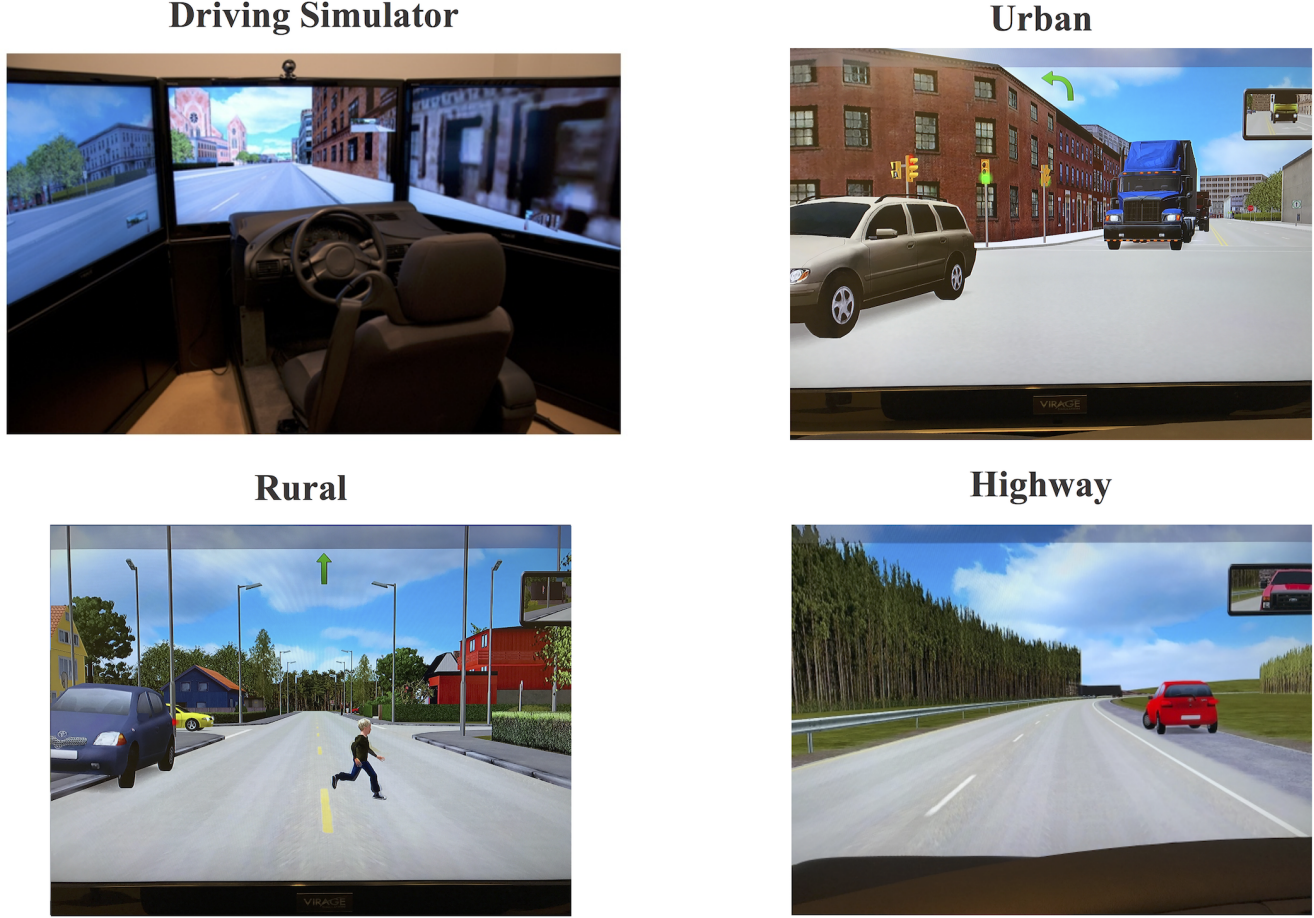
Image of the VS500M driving simulator and example of events belonging to the single visible conflict category within the Urban, Rural and Highway scenario. The event displayed on the top right panel (*i*.*e*. Urban scenario) corresponds to a cyclist violating the red light. During the events displayed in the bottom left panel (*i*.*e*. Rural scenario) and in the bottom right panel (*i*.*e*. Highway scenario) a car gets out of the drive-way and comes into the trajectory of the participant's car. These three events differed slightly to avoid participants’ anticipation but belong to the same category of events. Each panel corresponds to a photograph of the simulator’s central screen.

The second session included the assessment of subjects’ perceptual-cognitive skills with a 3-dimensional multiple object-tracking task (the 3D-MOT) adapted from Pylyshyn & Storm [33]. We implemented this test using a technology known as the NeuroTracker^TM^ (CogniSens) to assess the speed at which our participants could simultaneously track and attend multiple moving objects [34]. Next, participants were tested on the three distinct simulator scenarios, each representing different driving environments with different mental workload. Importantly, in each scenario, participants were instructed to drive as they normally would and follow visual and oral navigational instructions while respecting road signage, other road users and posted speed limits (*i*.*e*. 50 km/h in urban, 70, 90 km/h in rural and 100 km/h in highway scenario). In addition, to elicit their natural driving speed selections, no instructions were given about maintaining minimal speeds. Each of the three scenarios contained five to seven different skill testing, often dangerous, events that forced participants to respond (see **Fig 1**) and that were triggered at pre-programmed moments along the route. These events were homogenized across the three scenarios following the typology presented by Borowsky & Oron-Gilad [40] in their study on hazard perception. Each scenario incorporated both single-phased (i.e. hazard is always visible) and two-phased materialized hazards (i.e. hazard is hidden before becoming visible) that were either other vehicles or pedestrians and which required specific evasive responses and/or a sudden brake to navigate through safely. Participants’ results were averaged across all events for each scenario in order to provide large enough samples of their driving behaviour from which to conduct subsequent analyses.

### 2.4 Driving measures

To capture subtle changes in driving behavior between the three age groups, driving performance was evaluated using 18 specific driving measures (see **Table 1**). Driving is an inherently multifactorial task and variations in driving ability cannot necessarily be well understood based on any single measure or combination of different measures [41]. Therefore, rather than exclude potentially interesting measures based on subjective criteria, we developed a methodology aimed at better capturing the nuanced driving behavior of our subjects while also reducing our dataset to the most pertinent measures. Thus, all the measures recorded by the driving simulator were initially involved in our analyses. As a preliminary step, we performed a bivariate correlation on these 18 measures aggregated across the three scenarios. Based on this analysis, we controlled for the influence from varying mean driving speed and excluded variables correlated with this measure from further analyses. This preliminary step allowed us to exclude redundant and irrelevant information based on objective criteria.

**Table 1:** Definition of the studied measures and units in which they were recorded. n corresponds to an undefined unity, m to meters, s to seconds, km to kilometers, h to hours and log to logarithm.

	Measure	Unit	Description
1	**Crash**	n	Whether a collision occurred or not during the event.
2	**Near crash**	n	When within an event: - Subject brakes harder than a given threshold (0.7 for the rural scenario, 0.75 for other scenarios) while driving at a speed greater than 5 m/s (18km/h) - The steering wheel is turned more than 60 degrees while driving faster than a speed threshold (5 m/s) - The participant drives within 3 m of an object while travelling at a speed greater than 10m/s (36km/h) for the rural scenario, 5 m/s for other scenarios)
3	**Mean speed**	km/h	Average speed of all driving. For each data point, speed inferior to 10 km/h or recorded 300 m before and 100 m after an event were discarded from the averaging.
4	**SDLP**	m	Standard deviation of lateral position. Same exclusion criteria as mean driving speed computation were used. Additionally, for each data point, lateral position recorded 10 seconds before and after a lane changing were discarded from the averaging.
5	**Max brake**	n	Hardest amount of braking applied during event of interest. Where 0 = no braking applied, 1 = pedal is fully depressed.
6	**Distance at max brake**	m	Distance from object at which “Max brake” is recorded
7	**Max steer change rate**	degrees / s	Most extreme (in terms of range and speed) left or right steering wheel position change during event of interest.
8	**Distance at max steer change rate**	m	Distance for the most extreme (in terms of range and speed) left or right steering wheel position change during event of interest
9	**Steer range**	degrees	Difference in degrees between leftmost and rightmost steering wheel position for event of interest.
10	**Closest distance**	m	Minimum distance between participants’ vehicle and object during event
11	**Speed at closest**	m/s	Speed at which car is travelling when at minimum distance between participant and object during event.
12	**Hazard rating**	log (m/s/m)	Log of “Speed at closest” divided by the minimum distance between the participant's vehicle and the object. If there is a crash, this is computed from the last data point prior to the crash.
13	**Gas release speed**	m/s	Speed of vehicle at point when gas pedal is released for event of interest.
14	**Gas release distance**	m	Distance from object during event of interest when gas pedal is released.
15	**Brake speed**	m/s	Speed at which brake pedal is pressed during event of interest.
16	**Brake distance**	m	Distance from object at which brake pedal is pressed during event of interest.
17	**Speed at anticipation**	m/s	Speed at which vehicle starts decelerating for a minimum of 3 seconds for event of interest.
18	**Anticipation distance**	m	Distance from object at which vehicle starts decelerating for a minimum of 3 seconds for event of interest.

Four of the 18 selected measures have been widely used in driving simulator studies: “Crash”; “Near crash”; “Mean speed” that reflects a compensation strategy for age-related increases in response time [2] and standard deviation of lateral position (“SDLP”), which has been shown to be a sensitive measure of driver impairment [16, 42].

The remaining 14 measures are found less often in the literature but were selected for their potential to provide useful information. Notably, measures relying on the abrupt or unnecessary actions taken on the vehicle such as “Max brake”, “Distance at max brake”, “Max steer change rate”, “Distance at max steer change rate” and “Steer range” (see **Table 1**for a further description) might reveal poorly adapted and thus potentially risky behavior. Additionally, some of the measures reflect different responses adopted while facing hazardous events. The following are examples of measures that were taken during each event of interest: the distance and mean speed at which the gas pedal was released (“Gas release speed” and “Gas release distance”); the distance and mean speed at which brake pedal was pressed (“Brake speed” and “Brake distance”), and: the instant that the vehicle started decelerating for a minimum of three seconds (“Speed at anticipation” and “Anticipation distance”). The above listed measures were triggered at “event onset”, which is defined by a pre-programmed trigger occurring when a conflicting object enters the driver’s cone of vision (an invisible ‘cone’ traced in front of the vehicle that represents the visual information available to the forward-facing driver). Events were considered complete after the driver had proceeded through the scene and the object of interest was outside the cone of vision, regardless of participants’ reactions or event outcome. Apart from Mean speed and SDLP (which were computed along the entire scenario), the other 16 measures corresponded to the mean of values recorded on each event belonging to the same scenario.

### 2.5 Statistical analysis

Initially, bivariate correlations using the Pearson method were used to assess the relationship between the 18 driving measures aggregated across the three scenarios. A preliminary check of our data revealed that some variables violated the assumption of normality. Thus, non-parametric Spearman correlations were conducted for these variables.

Secondly, bivariate correlations were conducted on a scenario-by-scenario basis for variables that did not correlate with mean driving speed in our previous analysis. Partial correlations controlling for mean speed were employed to account for our decision to allow participants full control over their driving speed. Additionally, to investigate the differences in driving performance between age groups, Analyses of Covariance (ANCOVAs) controlling for mean speed was also conducted with these measures for each scenario. Parametric ANOVA was conducted on any dependent variables that did not significantly correlate with the covariate, (*i*.*e*., ‘mean driving speed’). In each case, post-hoc comparisons using Tukey’s HSD test were performed to explore differences between age groups. Non-parametric bootstrap-based ANOVA was performed on the variables that were non-normally distributed (number of iterations = 1000). This robust statistical method maintains the Type I error rate of the tests at its nominal level while also maintaining the power of the tests, even when the data are heteroscedastic and do not show normal distributions [43, 44]. In this specific case, multiple pairwise comparison procedures using the bias-adjusted percentile bootstrap method [45] were performed to explore differences between age groups.

Finally, to investigate how the scores obtained in the perceptual-cognitive task predict driving performance in relation to mental workload levels, we performed bivariate correlations between the scores obtained in the 3D-MOT task and the driving measures. 3D-MOT scores were measured using mean speed thresholds as the dependent variable and were computed based on the last four reversals of a 1-up 1-down staircase procedure with thirty trials. Correct or incorrect responses on each trial resulted in a proportional speed increase or decrease of 0.05 log units, respectively. Given the distribution of such psychophysical data, a logarithmic transformation was applied to the scores to permit conducting bivariate correlations between those scores and the driving measures. It has been suggested that multiple object tracking is a task correlated with some measures of driving ability [35, 46]. Therefore, it is conceivable that 3D-MOT might be a better predictor of risky driving behaviour than age and naturally adopted mean driving speed. Thus, for each driving measure we performed multiple linear regression analyses with Age, 3D-MOT score and mean driving speed as predictors.

## 3. Results

### 3.1 Mean driving speed and relevance of the driving measures

**Fig 2:**
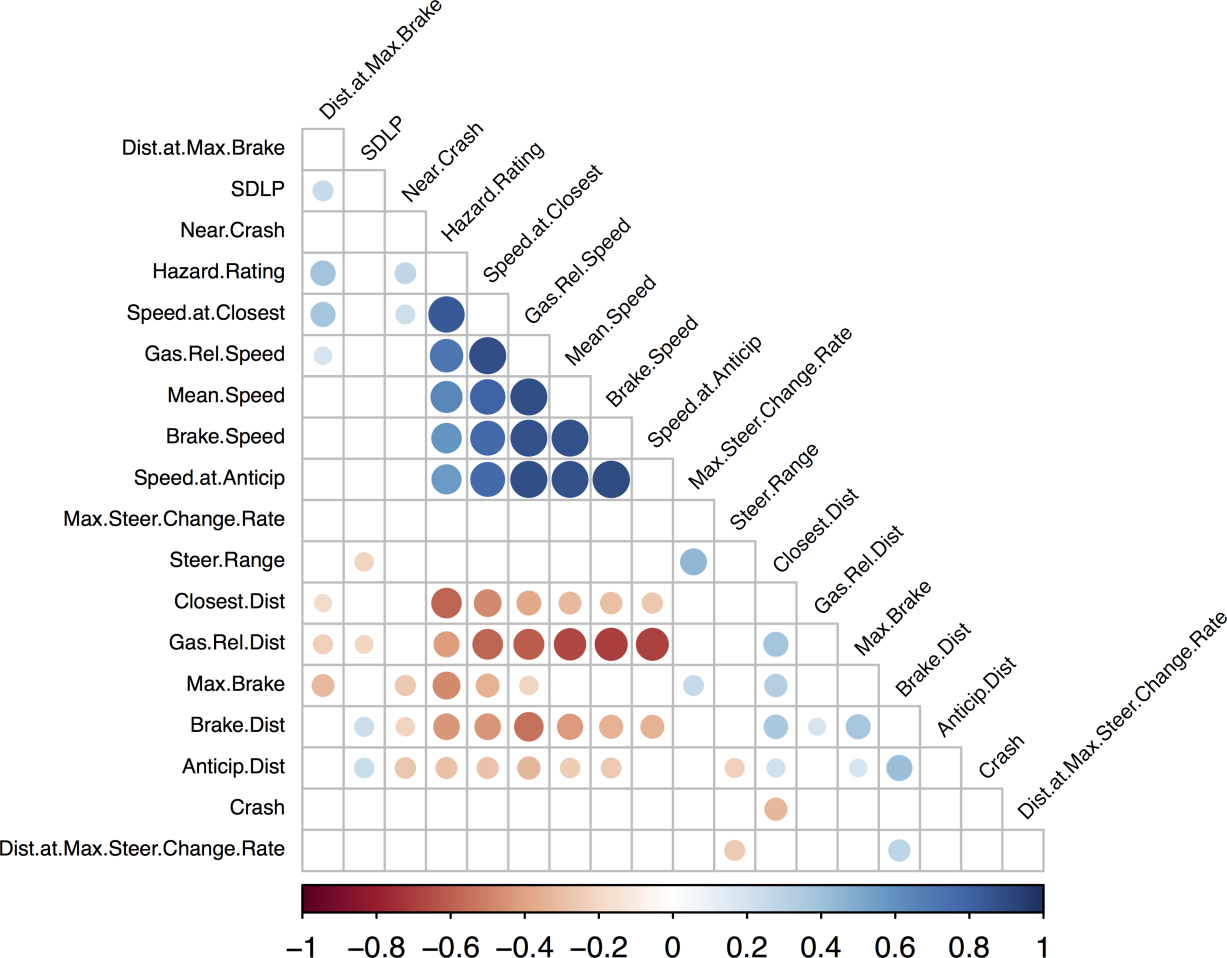
Graphical representation of the correlational analysis on the aggregated dataset performed using hierarchical clustering analysis in the R statistical environment (R Development Core Team, 2008). The size and color of each circle represents the magnitude and the direction of the correlation, respectively. Note that only the significant correlations (p< .05) appear on this graphical representation. As a striking result, the data can be clearly shown to be distributed into two clusters: one with positive correlations centered on Mean Speed and the other with negative correlations between speed measures and distance measures.

Pearson or Spearman correlation coefficients were computed between each driving measure. All the correlations were computed on values aggregated across the three scenarios and are summarized in **Fig 2**.

As expected, variables taking participants’ speed into account were very strongly correlated with mean driving speed. A hierarchical clustering analysis identified two clusters of variables: one centered around mean speed and including positive correlations, and the other with negative correlations between speed measures and distance measures (**Fig 2**). These latter relationships indicate that the higher the mean driving speed, the smaller the distances at which participants made their decisions regarding the event of interest. Thus, all these variables can be considered redundant and excluded from further analyses as they offer no additional/substantial information beyond the mean speed. Other measures such as Crash, Near crash, Standard deviation of lateral position (SDLP), Max steer change rate, Distance at max steer change rate, Max brake, Distance at max brake and Steer range were found to be independent of excessive mean speed influence and, as such, may provide relevant information for by-scenario analyses.

### 3.2. Influence of mental workload on driving measures

#### 3.2.1. Urban scenario

**Fig 3:**
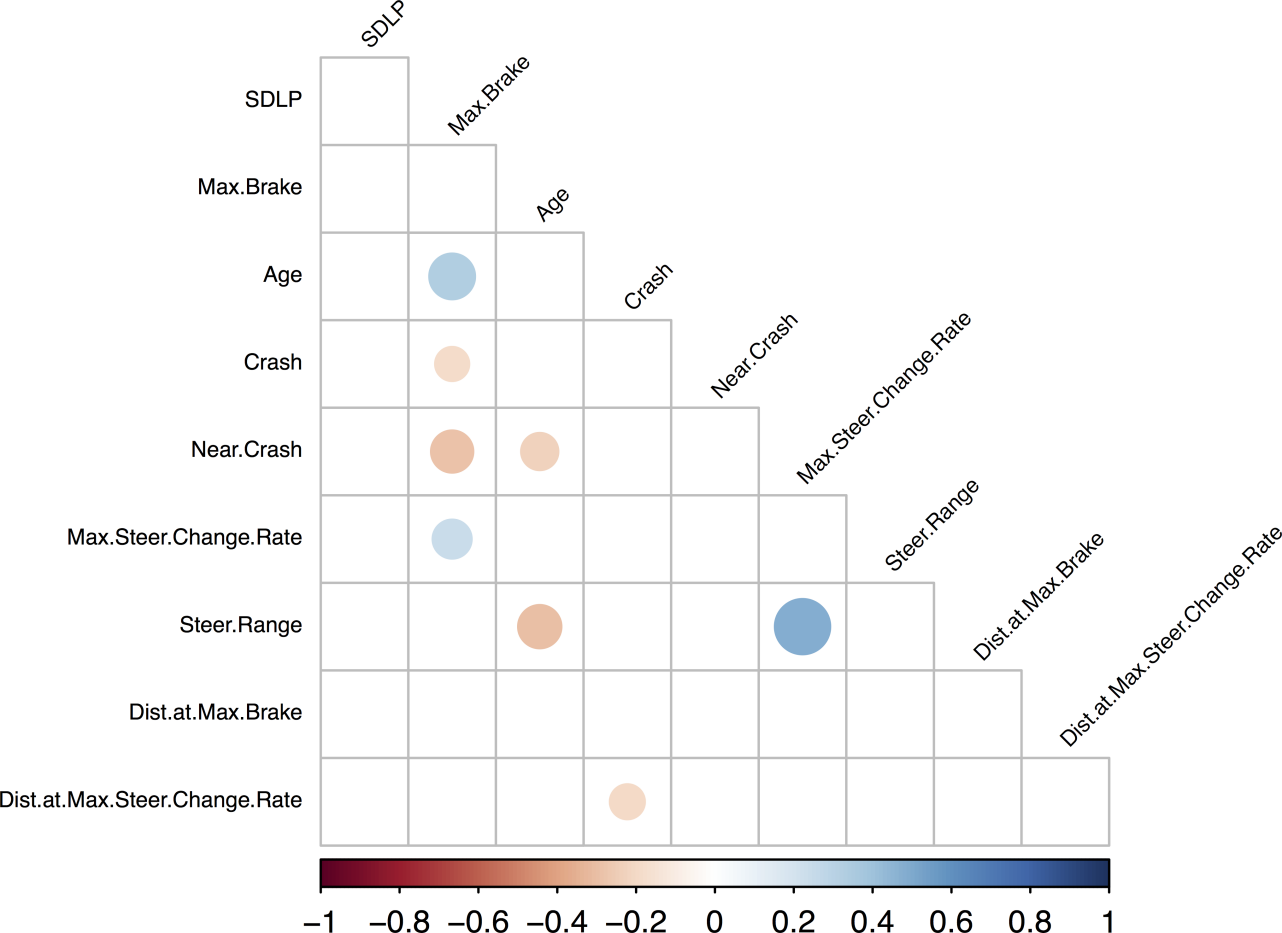
Graphical representation of the hcluster correlation analysis on the ‘Urban Scenario’ dataset controlling for mean speed. Only the significant correlations (p< .05) appear on this graphical representation.

We employed three scenarios of varying difficulty to determine which mental workload was the most appropriate to highlight subtle differences in driving behavior between drivers varying in experience. We first examined driving performances across age groups in the Urban scenario (designed to provide high challenge and thus high mental workload). Interestingly, correlations performed on measures recorded during this scenario showed that age was positively correlated with Max brake (r(115) = .33; p = < .001) and negatively correlated with Steer range (r(115) = —.3; p = < .001) and Near crash (r(115) = -.22; p = .019; see **Fig 3**). These correlations are corroborated by the ANCOVA which revealed significant age effects for Max brake (F(2,112) = 3.93; p = .02) and Steer range (F(2,111) = 4.36; p = .02) as well as a trend for Near crash (F(2,111) = 2.07; p = 0.06; **Table 2**). Multiple pairwise comparisons revealed that only the oldest group differed significantly on these measures compared to both inexperienced (Max brake: p = .06; Steer range: p = .03; Near crash p = .02) and experienced younger adults (Max brake = p = .05; Steer range p = .03; Near crash p = .03). There was no significant difference for these measures between the inexperienced and experienced groups (Max brake: p = .99; Steer range: p = .88; Near crash p = .61).

**Table 2:** Statistical age groups comparisons between the three age groups. When mean speed was correlated with the driving measure considered, an ANCOVA controlling for mean speed was used. When mean speed appeared to be uncorrelated with the driving measure considered, a parametric ANOVA was used. For non-normally distributed driving measures bootstrap-based ANOVA were used. The p-values resulting from the pairwise comparisons between Inexperienced, Experienced and Older drivers are shown on the three most right columns. For comparisons showing a significant difference or a strong tendency, an arrow indicates the direction of the difference.

*Measure*	Correlated With Mean Speed	Main Effect	Inexp. vs. Exp.	Inexp vs. Old	Exp. vs. Old
*Crash*	.096	.19	.25	.54	.17
*Near Crash*	< .001	.06	.61	**.02**	**.03**
*SDLP*	.32	.68	.94	.89	.66
*Max Brake*	.65	**.02**	.99	.06 (<)	.05 (<)
*Dist*. *at Max Brake*	.48	.89	.97	.89	.96
*Max Steer Change Rate*	.005	.26	.49	.94	.29
*Dist*. *at Max Steer Change Rate*	.002	.09	.54	.08	.32
*Steer Range*	.001	**.02**	.88	**.03 (>)**	**.03 (>)**
*Mean Speed*	x	**< .001**	.08	**.001 (>)**	**.001 (>)**

The above-mentioned results suggest that younger participants were more likely to experience near crashes than older participants. Statistical analyses also revealed that inexperienced (mean = 40.05; SD = ± 5.89) and experienced young drivers (37.26 ± 4.86) drove faster than older drivers (30.22 ± 4.86; both p = < .001). Although the statistical analyses were designed to control for mean driving speed, the slower driving speed exhibited by older participants is well-documented [2] and might explain the trend toward an association between younger age and higher near crash risk. Inexperienced drivers also tended to drive faster compared to experienced drivers (p = .08) in this scenario (**Table 2**). This tendency is consistent with previous findings showing that inexperienced drivers tend to take more risks while driving [15] and that higher speeds are associated with increased crash risk [47].

A possible interpretation of the positive correlation between age and Max brake might be that older participants were more likely to make abrupt stops. Additionally, it is possible that younger drivers may put themselves at greater risk for crashes by not braking hard enough during critical events. However, this latter result coupled with the negative correlation between Max brake and Crash (r(115) = -.18; p = .049) and the non-significant correlation between Age and Crash (r(115) = -.08; p = .39) suggests that younger participants may have adopted a smoother driving style than older participants without putting themselves at greater risk. The negative correlation between age and Steer range and the age effect on Steer range in the ANCOVA also suggests that older participants made fewer steering movements than younger participants during events of interest. Together, these results suggest two different types of avoidance strategies as a function of age younger drivers tended to favor steering movements to avoid crashes and older drivers were more likely to use abrupt braking strategies.

#### 3.2.2. Highway scenario

**Fig 4:**
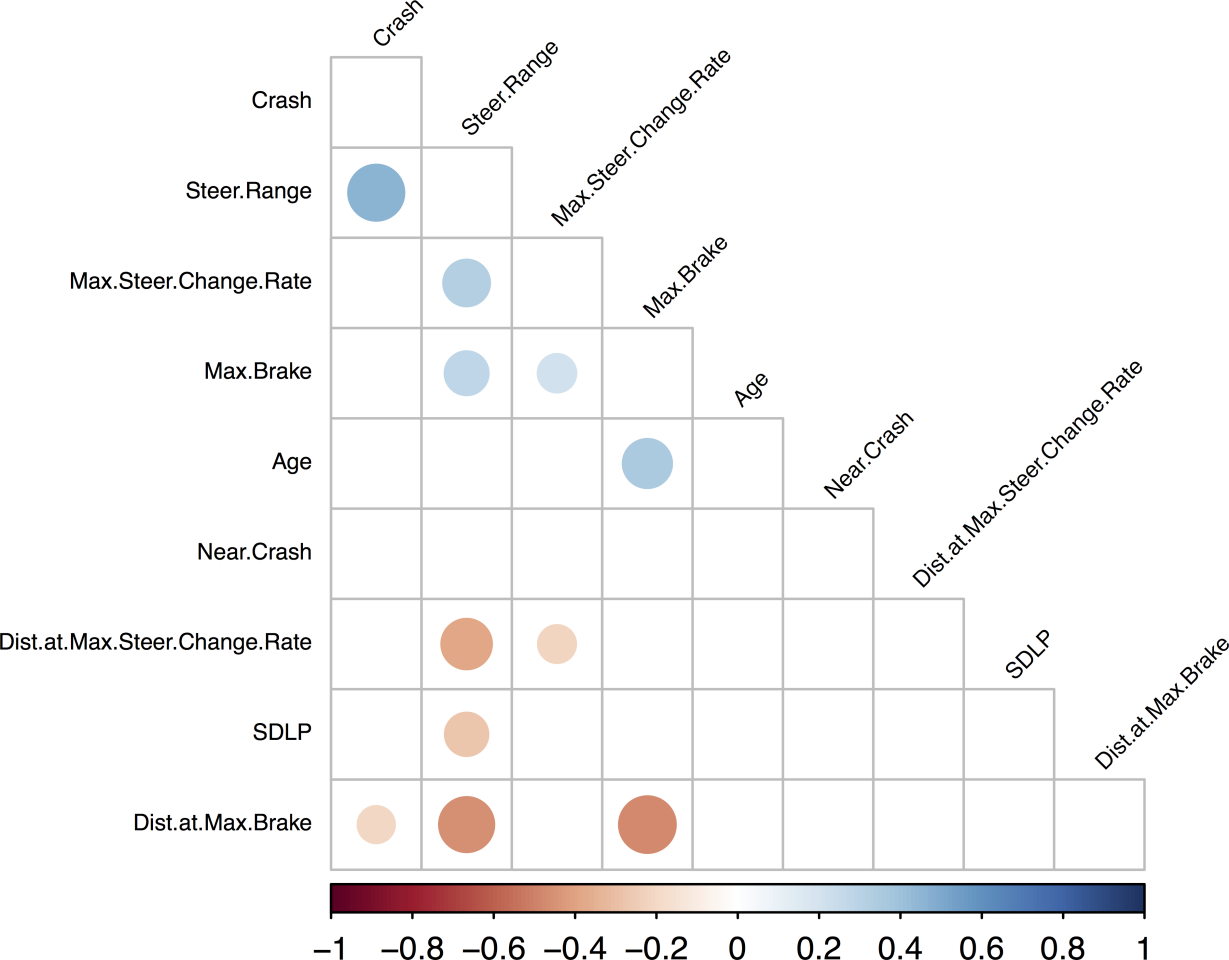
Graphical representation of the hcluster correlation analysis computed in R on the ‘Highway scenario’ dataset controlling for mean speed. Only the significant correlations (p< .05) appear on this graphical representation.

Contrary to the previous scenario, the ‘Highway’ route was designed to create a low mental workload. Strikingly, statistical analyses revealed that only two measures were dependent on age in this scenario. Indeed, we observed a positive correlation between age and Max brake (r(115) = .36; p < .001; **Fig 4**) as well as a significant age effect for Max brake (F(2,111) = 7.74; p < .001) and Mean speed (F(2,112) = 32.84; p < .001; **Table 3**). Multiple pairwise comparisons showed the same trend regarding the age effect on mean speed as the Urban scenario: Inexperienced (mean = 83.07 km/h; SD = ± 5.49) as well as experienced young drivers (77.93 ± 10.97) drove significantly faster than older drivers did (64.33 ± 12.69; all p < .001). An identical pattern emerged for Max brake, with the oldest participants also being far more likely to make full stops compared to both inexperienced (p = .003) and experienced young participants (p = .002). Slower speeds observed among older participants are unlikely to be related to the difficulty of this scenario, given that it was designed to be low in complexity. Instead, it is more likely that—in the absence of any external pressure to drive more quickly—older participants simply selected to drive at slower speeds than younger participants [48]. It can also be noted that despite the non-significant main effect of Age on Near crash [F(2,112) = 1.55; p = .19), multiple pairwise comparisons suggested that inexperienced drivers tended to incur more near crashes than experienced (p = .07) and older drivers (p = .06) in this scenario. This tendency might be linked to the well-documented propensity of young and inexperienced drivers to make riskier driving maneuvers [49, 50].

**Table 3:** Statistical age groups comparisons between the three age groups during the Highway scenario. When mean speed was correlated with the driving measure considered, an ANCOVA controlling for mean speed was used. When mean speed appeared to be uncorrelated with the driving measure considered, an ANOVA was used. For non-normally distributed driving measures bootstrap-based ANOVA were used. The p-values resulting from the pairwise comparisons between Inexperienced, Experienced and Older drivers are shown on the three most right columns. For comparisons showing a significant difference or a strong tendency, an arrow indicates the direction of the difference.

*Measure*	Correlated With Mean Speed	Main Effect	Inexp. vs. Exp.	Inexp vs. Old	Exp. vs. Old
*Crash*	.66	.25	.51	.5	.16
*Near Crash*	.17	.19	.07	.06	.96
*SDLP*	.053	.09	.09	.21	.97
*Max Brake*	.01	**< .001**	.96	**.003 (<)**	**.002 (<)**
*Dist*. *at Max Brake*	.007	.31	.93	.57	.28
*Max Steer Change Rate*	.04	.1	.98	.24	.1
*Dist*. *at Max Steer Change Rate*	.09	.07	.49	.06	.49
*Steer Range*	< .001	.39	.67	.92	.39
*Mean Speed*	x	**< .001**	.14	**.001 (>)**	**.001 (>)**

Rather than evidencing known age effects on measures such as SDLP [F(2,112) = 2.42; p = .09] and Crash [F(2,112) = 1.48; p = .25] [51], the present statistical analyses indicate that Steer range was a particularly interesting parameter in this scenario. Indeed, this measure was positively correlated with Crash (r(115) = .47; p < .001), Max brake (r(115) = .29; p = .002) and Max steer change rate (r(115) = .33; p < .001), and negatively correlated with SDLP (r(115) = -.28; p = .003), Distance at max brake (r(115) = -.45; p < .001) and Distance at max steer change rate (r(115) = -.38; p < .001). The pattern suggests that Distance at max steer change rate might be an important parameter to consider in this scenario. The correlation between Steer range and Max steer change rate as well as Max brake indicates compensatory actions by drivers facing risky situations, as outlined by Pacaux-Lemoine et al. [52]. The negative correlations between Steer range and both Distance at max steer change rate as well as Distance at max brake further reflect these compensatory actions. More revealing, however, is the negative correlation between ‘Steer range’ and ‘SDLP’. While both measures seem intuitively related, it is important to consider that ‘Steer range’ is a measure of the absolute difference between the leftmost and rightmost steering wheel position and SDLP is related to variability in lane position. One might expect a positive correlation between these measures if variability in SDLP scores was merely related to variability introduced by one instance of extreme steering wheel action taken at the last second before a crash. Instead, the negative relationship points to greater lane position variability in individuals who did not eventually make extreme steering adjustments and thus exhibited greater vehicle control and were presumably at lower crash risk. This result is somewhat inconsistent with the body of literature suggesting that higher SDLP is associated with decreased vehicular control [53].

#### 3.2.3. Rural scenario

**Fig 5:**
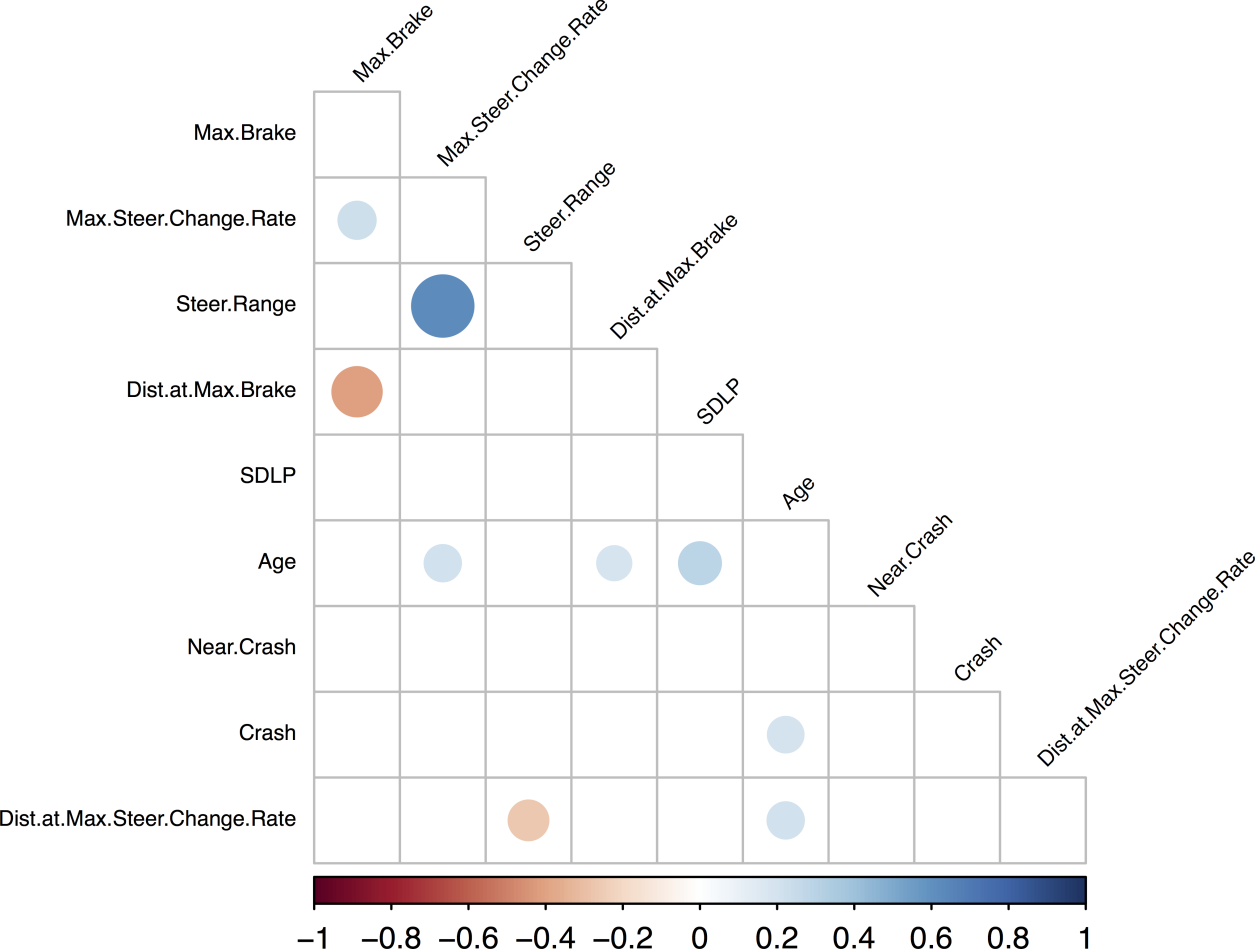
Graphical representation of the hcluster correlation analysis computed in R on the ‘Rural Scenario’ dataset controlling for mean speed. Only the significant correlations (p< .05) appear on this graphical representation.

The last of the three scenarios, the Rural scenario, was designed to produce a moderate mental workload. Contrary to the other two scenarios, here ‘Age’ was the measure most strongly correlated with various driving measures. Indeed, partial correlations (**Fig 5**) showed a positive correlation between ‘Age’ and ‘Crash’ (r(115) = .21; p = .025), ‘SDLP’ (r(115) = .29; p = .002), ‘Max steer change rate’ (r(115) = .22; p = .019), ‘Distance at max steer change rate’ (r(115) = .22; p = .02), ‘Distance at max brake’ (r(115) = .19; p = .04) as well as a strong tendency for the correlation between ‘Age’ and ‘Max brake’ (r(115) = .18; p = .056). Statistical comparisons between the three age groups (**Table 4**) corroborate some of these interrelationships by showing that Age was a determinant factor for crash occurrence [F(1,112) = 3.55; p = .03] and SDLP [F(1,112) = 4.86; p = .009]. The propensity of older adults to be more involved in crashes and to show larger SDLP under increased cognitive load has been previously demonstrated [54] and linked to age-related deficits in certain driving skills [55–57]. Nevertheless, the important result here is that the rural scenario was the only one to reveal these age differences, suggesting that the mental workload involved in this scenario might be the most efficient method for detecting subtle age differences.

**Table 4:** Statistical comparison between the three age groups during the Rural scenario. When mean speed was correlated with the driving measure considered, an ANCOVA controlling for mean speed was used. When mean speed appeared to be uncorrelated with the driving measure considered, an ANOVA was used. For non-normally distributed driving measures bootstrap-based ANOVA were used. The p-values resulting from the pairwise comparisons between Unexperienced, Experienced and Older drivers are shown on the three most right columns. For comparisons evidencing a significant difference, an arrow indicates the direction of the difference.

*Measure*	Correlated With Mean Speed	Main Effect	Inexp. vs. Exp.	Inexp vs. Old	Exp. vs. Old
*Crash*	.08	**.03**	.49	**.047 (<)**	**.008 (<)**
*Near Crash*	.63	.32	.27	.98	.25
*SDLP*	.93	**.009**	.42	**.008 (<)**	.17
*Max Brake*	.71	.06	.77	.33	.06
*Dist*. *at Max Brake*	.07	.49	.92	.78	.47
*Max Steer Change Rate*	.49	.16	.96	.21	.29
*Dist*. *at Max Steer Change Rate*	.42	**.02**	.1	**.02 (<)**	.82
*Steer Range*	.03	.71	.99	.8	.73
*Mean Speed*	x	**< .001**	.76	**.001 (>)**	**.003 (>)**

Interestingly, multiple pairwise comparisons showed that older drivers were significantly more likely to have a crash on this route compared to both inexperienced (p = .047) and experienced drivers (p = .008). The breakdown of the main age effect of ‘SDLP’ revealed that older drivers showed significantly greater SDLP than inexperienced drivers (p = .008) but there was no significant difference with experienced drivers (p = .17). The same pattern of results was observed in ‘Distance at max steer change rate’. While the one-way ANOVA showed a significant age effect [F(1,112) = 4.03 p = .02], multiple pairwise comparisons demonstrate that this effect was mainly attributable to the significant difference between older and inexperienced drivers (p = .02). There was no significant difference between older and experienced drivers (p = .82) nor between inexperienced and experiences drivers (p = .1). The lack of significant differences in driving behaviors between inexperienced and experienced drivers might reflect the fact that even relatively inexperienced drivers are capable of quickly learning basic vehicle maneuvering and traffic situations while still lacking the higher-order skills and motivations necessary to be safe in the wide variety of contexts seen in real-world driving (see Hatakka et. al. [10] for a review). Thus, the performance deficit observed in older drivers is also unlikely to be related to basic driving skills and may reflect greater sensitivity to increased task demands. Ultimately, these results reinforce the idea that performance on the rural scenario might be the most sensitive to subtle differences in driving behaviors.

Finally, while one-way ANOVA again revealed a significant age effect on the mean driving speed on this scenario (F(2, 112) = 10.01, p < .001), the quantitative differences in mean speeds of the three groups (inexperienced: 73.81 ± 6.6; experienced: 72.21 ± 7.24; older: 65.45 ± 11.04) actually represent smaller percent differences (12% between the slowest and fastest groups) compared to the Urban (28%) and Highway (25.43%) scenarios. The relative equalization of mean speed observed may thus position the moderate complexity Rural scenario as a particularly valid representation of naturalistic group differences in behavior when faced with hazardous driving events.

Taken together, these findings suggest that after perceiving potential threats, older drivers took defensive measures earlier than younger drivers but were also less likely to identify these threats in sufficient time to react appropriately. This pattern might be related to slowed and altered motor responses among older individuals [58, 59] but may also be linked to perceptual-cognitive changes [7, 60] associated with ageing. While the nature of such changes has been studied extensively, researchers have only recently explored their implications for driving safety [35, 44].

### 3.3. Perceptual-cognitive measures

The use of different scenarios reveals several subtle differences in the driving performance of our three age and experience groups. Critically, the identification of these differences seems to require driving scenarios with appropriate levels of difficulty and mental workload (i.e. the Rural scenario). The main age difference was observed in the control of vehicle speed with older participants driving more slowly than younger ones. Nevertheless, current driving measures are not sufficient to explain these large differences in mean speed. Additionally, except for the mean speed measure, age differences were not indicative of other changes in vehicle control measures. Instead, differences may reflect how older individuals process and respond to upcoming events.

**Fig 6:**
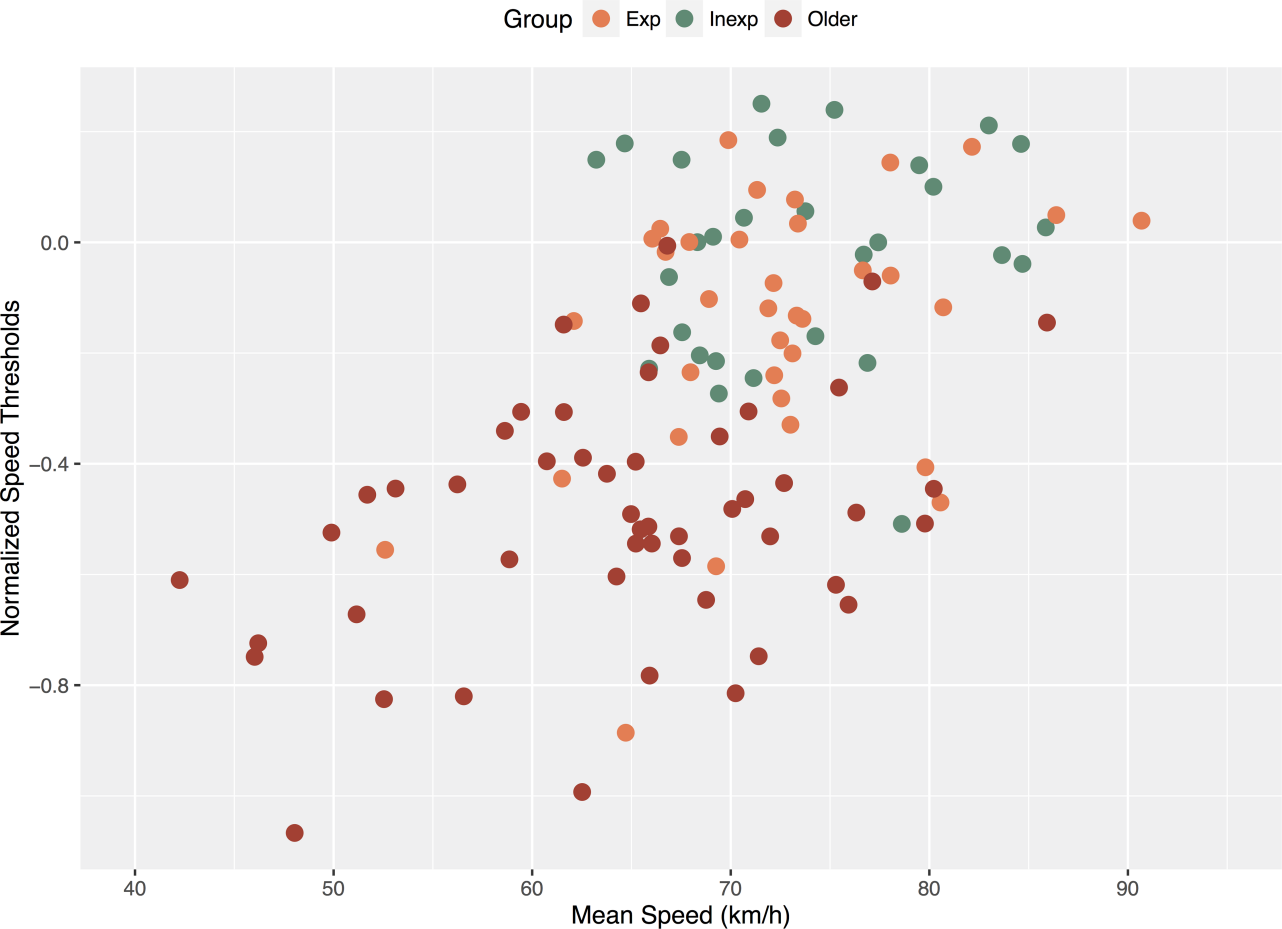
Correlation between NeuroTracker speed thresholds (represented in log units) and mean speeds naturally adopted in the rural scenario.

To investigate this idea, we turned to the NeuroTracker measures and examined bivariate correlations between 3D-MOT scores and driving measures recorded during the Rural scenario (i.e. the scenario showing the greatest age differences). Strikingly, statistical analysis revealed that 3D-MOT scores were significantly correlated with ‘Crash’ (*r*(113) = -.31, p < .001), ‘SDLP’ (*r*(113) = -.26, p < .005), Distance at Max Steer Change Rate (*r*(113) = -.2 p = .03) and Mean Speed (*r*(113) = .47, p < .001) measures (**Table 5 and Fig 6**). These results show that the more the perceptual-cognitive abilities were altered (as evidenced through NeuroTracker speed thresholds), the more driving speed decreased and crash occurrence increased. These findings are consistent with the hypothesis that the low mean speed observed in older people is linked to a self-restriction due to the alterations of their perceptual-cognitive abilities. A decrease in these abilities has been previously linked to crash risk (see Anstey et. al. [61] for an in-depth review). While past research has focused on more isolated perceptual-cognitive factors (i.e. selective and divided attention, processing speed, useful field of view, etc.), to date comparatively little research has made use of more integrative and dynamic tests like the NeuroTracker. If decreased perceptual-cognitive ability is indeed related to driving performance, then we should expect performance on the NeuroTracker to be associated with of our driving measures.

**Table 5:** The 3D-MOT as a predictor of a risky driving behavior. Bivariate correlations between perceptual-cognitive measure and driving measures across the three scenarios.

*Measure* (Rural)	*R*	*p*
*Crash*	-.31	**< .001**
*Near Crash*	-.04	.65
*SDLP*	-.26	**.005**
*Max Brake*	-.01	.92
*Dist*. *at Max Brake*	.-.07	.45
*Max Steer Change Rate*	-.07	.47
*Dist*. *at Max Steer Change Rate*	-.2	.03
*Steer Range*	.16	.09
*Mean Speed*	.47	**< .001**

We subsequently performed a multiple linear regression analysis on these data to model the extent to which these driving measures could be predicted by Neurotracker speed thresholds, age and mean driving speed. These latter two measures were included within the model as additional predictors to clarify the relative value of each component. The model significantly predicts crash [F(3,111) = 4.53; p = .005; R = .33], SDLP [F(3,111) = 4.37; p = .006; R = .32], Distance at max brake [F(3,111) = 3.06; p = .03; R = .28] and Mean speed [F(3,111) = 16.66; p < .001; R = .48] and shows a tendency to predict Max brake [F(3,111) = 2.36; p = .07; R = .25] as well as Distance at max steer change rate [F(3,111) = 2.26; p = .09; R = .24]. However, this model was non-significant for the measures Near crash [F(3,111) = 1.11; p = .35; R = .17], Max steer change rate [F(3,111) = 2.03; p = .11; R = .23] and Steer range [F(3,111) = 1.84; p = .15; R = .22]. Interestingly, NeuroTracker speed threshold was the only significant predictor of crashes (β = -.36; t = -2.75; p = .007) and was predictive of naturally adopted mean speed (β = .37; t = 3.18; p = .002) (**Table 6**). Additionally, whereas NeuroTracker speed threshold only shows a tendency to predict ‘Max brake’ (β = .23; t = 1.75; p = .08), age emerged as a significant predictor of ‘Max brake’ (β = .34; t = 2.61; p = .01). Finally, Mean speed predicted ‘Distance at max brake’ (β = .3; t = 2.83; p = .006). Such results are consistent with recent results from MacKenzie & Harris [35] who also demonstrated the usefulness of MOT and measures of attentional resources in predicting aspects of driving performance as well as more pronounced effects related to road complexity. Thus, our findings support the growing research consensus that age-group related differences in driving behaviors are associated with measurable changes in underlying perceptual-cognitive abilities [35, 62, 63].

**Table 6:** The 3D-MOT as a predictor of a risky driving behavior. **Multiple linear regression analyses performed on measures recorded during the rural scenario.** 3D-MOT scores, Age and Mean driving speed were entered as predictors in the model. For each driving measure, regression weights (β) and significance value (p) are shown.

*Predictor*		Crash	Near Crash	SDLP	Max Brake	Dist. at Max Brake	Max Steer Chg. Rate	Dist.at Max Steer Chg. Rate	Steer Range	Mean Speed
**LogNT**	*β*	-.36	-.11	-.21	.23	-.11	.02	-.1	.04	.37
	*p*	**.007**	.42	.12	.08	.4	.89	.47	.74	**.002**
**Age**	*β*	.02	-.17	.2	.34	.14	.25	.18	-.06	-.15
	*p*	.87	.2	.13	**.01**	.28	.06	.17	.63	.21
**Mean Speed**	*β*	.13	-.14	.17	-.02	.3	.16	.04	.15	x
	*p*	.2	.2	.11	.85	**.006**	.14	.7	.15	x

## 4. Discussion

### 4.1 Mental workload and driving measures

The main aim of the present study was to determine the degree to which scenario complexity produces an appropriate level of mental workload for the dual purpose of: (1) eliciting realistic behavior in challenging circumstances and (2) revealing subtle differences in driving ability across a wide-range of different age and experience groups. Numerous studies confirm the existence of differences in driving behavior both on-road and in a driving simulator between age groups and levels of experience [56, 64–67]. By manipulating the situation complexity in three distinct simulator scenarios, we showed that reliably identifying said differences in cross-sectional research seems to require a scenario designed with a moderate level of difficulty and workload. Indeed, only one scenario, the Rural, exhibited large correlations between age and well-known driving measures such as crash occurrence, a clear negative outcome, and SDLP, a sensitive measure of driver impairment [68]. The limited age effect on driving measures observed in our Urban scenario (i.e. high demand) suggests that the scenario’s increased mental workload and slower required driving speed may have homogenized participants’ reactions and behavior enough to mask subtler differences between how different age groups respond to challenging driving events. A few differences were found in the types of potentially risky driving behavior exhibited by different age groups, i.e. in measures linked to a performance deficit such as near crashes and in the differences in danger avoidance strategies. Similar outcomes were found for the Highway (low demand) scenario.

A better understanding of the optimal mental workload for testing differences between age groups in driving simulator studies is of social interest because it has been recognized that mental workload related problems are responsible for most road accidents [69]. Given that the cognitive capacity of the human brain is limited [28, 70], that aging is associated with decreased cognitive capacity [54, 71] and that task performance can be impaired when the resource demands exceed resource availability [27], one might expect a close relationship between increased age, scenario complexity and poor driving performance. Not all our results indicated an association between factors related to age and scenario complexity. Some of these agree with the models proposed by Meister [72] and de Waard [16] that assume that in cases of either extreme high or low task demands, drivers can become overloaded or under aroused and thus task measures may lose some sensitivity. Thus, the similar results observed between the high-complexity Urban scenario and the low-complexity Highway scenario might be in-part explained by decreased vigilance during the low-demand scenario resulting in a higher mental workload for the events themselves [16]. A review of the literature done by Paxion et. al. [36] has made similar suggestions in this regard. This interpretation, indicating an “inverse-U” model of the mental workload effects on performance, is corroborated by our finding that the Rural scenario was best at naturally reducing age group mean speed variability—another factor worthy of discussion in the context of cross-sectional driving simulator research.

### 4.2 A new outlook on mean driving speed

We did not impose a minimum driving speed on participants to gain insight into their natural driving behavior. One of the main findings of the present study is the degree to which drivers of different age groups naturally self-select their driving speeds. In the absence of external pressure to drive more quickly, older adult participants drove significantly slower than both inexperienced younger and experienced adult participants. Though slower driving speeds could be expected for older drivers [2], artificially slower speeds might be problematic in the context of driving studies that seek to measure reactions to dangerous events. Indeed, slower drivers would have more time to perceive and process upcoming threats and react appropriately.

This result already provides one important insight for studies investigating potentially risky driving behavior: While naturally-adopted mean speeds are informative, individuals’ driving speeds in the simulator should be somewhat controlled to better ensure that the task elicits ecological driving behavior for all participants. Moderate scenario complexity may be one method of naturally reducing mean speed variability between age groups as observed in the Rural scenario. Beyond that, other relatively unobtrusive solutions such as sensory feedback should be investigated to reduce the range of this variability without eliminating it entirely. Finally, given the well-documented decrease in visual processing speed of older adults [73], one could imagine that encouraging more equal driving speeds may further distinguish the driving performance of different age groups.

### 4.3 Novel measures of driving performance: Uncontrolled and abrupt maneuvers

Several novel driving metrics were conceived of and evaluated to determine their usefulness in quantifying driving performance in a more nuanced fashion than traditional measures such as mean speed, crashes and ‘SDLP’. Past research has suggested that driving simulator studies might be more sensitive to subtle changes in driving performance than on-road assessment [74]. Thus, such studies represent an interesting opportunity to measure these subtle differences using a diverse set of driving measures. While many of these measures were ultimately excluded based on strong correlations with mean driving speed, a few did emerge as significant, non-redundant measures of driving behavior. Notably, measures of the maximum amount of force pressed on the brake pedal during events of interest (‘Max brake’) as well as the range and speed of steering action participants exhibited (‘Max steer change rate’) emerged as potential indicators of risky or useless action taken upon the vehicle. In addition, the distance at which these actions were taken (‘Distance at max brake’ and ‘Distance at max steer change rate’, respectively) seemed to identify drivers who respond later to upcoming threats and who subsequently acted in extreme ways to avoid collisions, a finding consistent with descriptions by Pacaux-Lemoine et al. [52] of drivers forced into similar simulated circumstances. Although the current study design limits this possibility, it would be interesting to examine the effectiveness of a number of these driving metrics with imposed driving speeds.

### 4.4 Perceptual-cognitive ability predicts driving performance

The correlations observed between NeuroTracker speed thresholds and driving measures such as crashes, SDLP and mean speed in the rural scenario reinforce the notion that the slower mean driving speed of older people may in fact reflect compensatory behavior for changes in perceptual-cognitive ability. Our multiple linear regression model demonstrated that NeuroTracker speed thresholds were better at predicting many of aspects of driving behavior than naturally-adopted mean speed and age. Numerous studies have already linked tests of perceptual-cognitive abilities with changes in driving performance due to aging [75–77]. While previous studies have shown a relationship between MOT and some driving measures [35, 44], our results clearly reinforce and extend these findings to indicate that 3D-MOT is a relevant predictor of driving performances across age.

Older drivers have slower reaction times due to normal aging [78]. In addition, normal aging is associated with decrements in perceptual-cognitive abilities such as visual attention [7], visual processing speed [79] and working memory [80]. Many of the previously mentioned tests are designed to independently measure one of these facets of perception or cognition. While this is not inherently problematic, it represents a significant issue when trying to study a complex and visually demanding activity such as driving [60]. A recent study by Cuenen et. al. [22] has suggested that different aspects of driving behavior are better predicted by specific perceptual and cognitive “functional” abilities. 3D-MOT has proven to be an integrative measure of several of these different abilities [81]. This highlights not only the link between attentional function and driving but also suggests the importance of incorporating a dynamic assessment of sustained visual attention when studying driving performance.

A recent meta-analysis by Vanlaar et. al. [82] reports robust evidence that suggests that cognitive screening instruments have value in predicting driving ability, although at present there is no single instrument that can accurately identify an unsafe driver. The well-known test traditionally used to assess attention through the visual field and, thus, aspects of driver safety, is the Useful Field of View (UFOV). One of the main limitations of this test is that it does not directly assess dynamic processing, unlike MOT. Our results from the correlational and regression analysis suggest that NeuroTracker speed threshold measures may be comparable to results obtained by Cuenen et. al. [83] for UFOV in predicting specific aspects of driving ability (e.g. mean driving speed). This interpretation must be taken with caution, however, as one limitation of the current study is the lack of a UFOV performance measure. Future research should compare NeuroTracker thresholds more directly with the UFOV as well as other tests of perceptual-cognitive function to evaluate its effectiveness as a more integrative predictor of driving risk.

## 5. Conclusion

The present experiment was designed to determine the efficacy of different scenarios to elicit naturalistic driving behavior across different age groups. Insights from this study provide a justification for several different improvements of future cross-sectional driving research as well as new insights into how subtle changes in perceptual-cognitive abilities might impact specific aspects of driving behaviour. For instance, it highlights the need for study designs to include appropriate scenario selection and driving task complexity to reduce the variability of naturally adopted driving speeds. This finding provides a rationale for future research to use more overt methods (*i*.*e*. sensory feedback) to encourage participants to drive at more uniform mean speeds alongside statistical methods of controlling for such variability. Additionally, new driving behavior measures developed in this study may be used to account for inappropriate driver actions. Notably, higher ‘Max brake’ and ‘Max steer change rate’, as well as decreased distances from the hazards at which both of these extreme behaviours took place, emerged as possible indicators of at-risk driving. These novel measures of uncontrolled and abrupt driving maneuvers warrant further investigation and inclusion alongside more traditional measures of driving ability. Finally, perceptual-cognitive measures can help quantify the underlying factors of diminished driving performance—notably, helping to identify participants that might be engaging in compensatory driving behaviour but still at increased risk. Such a result is in line with established literature and provides an impetus for further study into how these mental faculties can be used both for identifying and possibly helping drivers with diminished driving safety.
